# Inhibition of Heme Export and/or Heme Synthesis Potentiates Metformin Anti-Proliferative Effect on Cancer Cell Lines

**DOI:** 10.3390/cancers14051230

**Published:** 2022-02-27

**Authors:** Anna Lucia Allocco, Francesca Bertino, Sara Petrillo, Deborah Chiabrando, Chiara Riganti, Alberto Bardelli, Fiorella Altruda, Veronica Fiorito, Emanuela Tolosano

**Affiliations:** 1 Molecular Biotechnology Center, Department of Biotechnology and Health Sciences, University of Torino, 10126 Torino, TO, Italy; annalucia.allocco@unito.it (A.L.A.); francesca.bertino@unito.it (F.B.); sara.petrillo@unito.it (S.P.); deborah.chiabrando@unito.it (D.C.); fiorella.altruda@unito.it (F.A.); emanuela.tolosano@unito.it (E.T.); 2 Department of Oncology, University of Torino, 10126 Torino, TO, Italy; chiara.riganti@unito.it; 3 Department of Oncology, University of Torino, 10060 Candiolo, TO, Italy; alberto.bardelli@unito.it; 4 Candiolo Cancer Institute, FPO-IRCCS, 10060 Candiolo, TO, Italy

**Keywords:** heme, cancer, FLVCR1, FLVCR1a, ALAS1, metformin, mitochondria, metabolism, therapy

## Abstract

**Simple Summary:**

Tumor initiation and progression are sustained by the ability of the cancer cell to reshape its metabolism in a way that favors cell proliferation and survival. Recently, it was shown that heme metabolism contributes to metabolic adaptation of tumor cell and that interfering with heme homeostasis reduces tumor cell growth. Here, we show that the alteration of heme metabolism, either by RNA-interference or pharmacological approaches, increases the sensitivity of tumor cell lines to the antitumor agent metformin. These findings strengthen the concept of targeting heme metabolism to counteract tumor progression.

**Abstract:**

Cancer is one of the leading causes of mortality worldwide. Beyond standard therapeutic options, whose effectiveness is often reduced by drug resistance, repurposing of the antidiabetic drug metformin appears promising. Heme metabolism plays a pivotal role in the control of metabolic adaptations that sustain cancer cell proliferation. Recently, we demonstrated the existence of a functional axis between the heme synthetic enzyme ALAS1 and the heme exporter FLVCR1a exploited by cancer cells to down-modulate oxidative metabolism. In colorectal cancer cell lines, the inhibition of heme synthesis-export system was associated with reduced proliferation and survival. Here, we aim to assess whether the inhibition of the heme synthesis-export system affects the sensitivity of colorectal cancer cells to metformin. Our data demonstrate that the inhibition of this system, either by blocking heme efflux with a *FLVCR1a* specific shRNA or by inhibiting heme synthesis with 5-aminolevulinic acid, improves metformin anti-proliferative effect on colorectal cancer cell lines. In addition, we demonstrated that the same effect can be obtained in other kinds of cancer cell lines. Our study provides an in vitro proof of concept of the possibility to target heme metabolism in association with metformin to counteract cancer cell growth.

## 1. Introduction

Heme, an iron-coordinating porphyrin, is indispensable for cells. When bound to proteins, it is involved in multiple biological processes, which encompass oxygen transport and storage [1], energy metabolism modulation [2] and gene expression regulation [3]. Nevertheless, free unbound heme is toxic for cells as heme-iron catalyzes the formation of reactive oxygen species (ROS), promoting tissue damage [3].

For this reason, heme levels are tightly regulated by the balance between heme biosynthesis, incorporation into hemoproteins, degradation and trafficking across cell membranes [3]. Heme biosynthesis starts in mitochondria with the 5-aminolevulinic acid synthase 1 (ALAS1)-catalyzed condensation of succinyl-CoA and glycine to produce 5-aminolevulinic acid (ALA). Subsequent enzymatic reactions form the porphyrin ring, which is bound to iron to generate heme. Heme can be incorporated into hemoproteins and heme excess can be degraded by heme oxygenases (HMOX 1 and 2) or exported out of the cell by two exporters expressed at the plasma membrane: the ATP-binding cassette sub-family G member 2 (ABCG2) [4] and the Feline Leukemia Virus subgroup C Receptor (FLVCR) 1a, the canonical isoform of *FLVCR1* gene [5].

We recently identified a functional link between heme synthesis and FLVCR1a-mediated heme export, in which heme efflux sustains heme synthesis by limiting heme-mediated inhibition of ALAS1 [6]. In cancer cell lines, the heme synthesis-export axis modulates oxidative metabolism by regulating the tricarboxylic acid (TCA) cycle flux. When the axis is enhanced by promoting heme efflux, TCA cycle is down-modulated and oxidative phosphorylation (OXPHOS) decreases. Conversely, when the axis is blocked by either inhibiting heme export or heme synthesis, TCA cycle and OXPHOS are enhanced. Interestingly, the metabolic alterations upon the blockage of this system result in decreased cell proliferation/survival. Based on these data, we proposed that the heme synthesis-export axis contributes to metabolic adaptations that sustain tumor cell growth.

Interfering with metabolic reprogramming of tumor cells represents a promising strategy to limit tumor growth and to increase sensitivity to pharmacological treatments. In this context, alterations of heme homeostasis were proved to be effective in several tumors. Heme dependent degradation of BACH1 restores metformin sensitivity in resistant triple negative breast tumors [7]. The inhibition of HMOX1 is lethal for fumarate hydratase deficient renal cancer cells and increases gemcitabine cytotoxic effect in pancreatic and urothelial cancer cells [8,9,10]. Furthermore, ALA administration improves artemisinin antitumoral effect in colorectal cancer [11,12]. Finally, heme biosynthesis down-modulation sensitizes Acute Myeloid Leukemia cells to apoptosis-inducing therapies [13].

Here, we aim to assess whether the modulation of the heme synthesis-export system affects the sensitivity of colorectal cancer (CRC) cells to pharmacological treatment. Our data demonstrate that the inhibition of ALAS1/FLVCR1a axis increases the sensitivity of CRC cells to metformin. Moreover, we demonstrate that the same effect can be obtained in other kinds of cancer cell lines, thus providing the rationale for the development of combined anti-tumoral therapies.

## 2. Materials and Methods

### 2.1. Cell Culture

SKCO1 (ATCC: HTB-39™) were propagated in Minimal essential medium (MEM, Gibco by Thermo Fisher Scientific, Waltham, MA, USA, catalog No. 21090022) supplemented with 10% heat-inactivated low-endotoxin FBS (Gibco by Thermofisher Scientific, Waltham, MA, USA, catalog No. 10270106) and 2 mM L-glutamine (Thermo Fisher Scientific, Waltham, MA, USA, catalog No. 25030024). CACO2 cells (ATCC: HTB-37™) were maintained in Dulbecco’s modified Eagle’s medium (DMEM, high glucose, GlutaMAX supplement; Gibco by Thermofisher Scientific, Waltham, MA, USA, catalog No. 61965059) supplemented with 20% heat-inactivated low-endotoxin fetal bovine serum (FBS; Gibco by Thermofisher Scientific, Waltham, MA, USA, catalog No. 10270106), 1 mM Sodium Pyruvate (Gibco by Thermofisher Scientific, Waltham, MA, USA, catalog No. 11360039) and 1X MEM Non-essential amino acids solution (Gibco by Thermofisher Scientific, Waltham, MA, USA, catalog No. 11140035). HPAC (CRL-2119™) and PANC (CRL-1469™) cells were propagated in Dulbecco’s modified Eagle’s medium (DMEM, high glucose, GlutaMAX supplement; Gibco by Thermofisher Scientific, Waltham, MA, USA, catalog No. 61965059) supplemented with 10% heat-inactivated low-endotoxin fetal bovine serum (FBS; Gibco by Thermofisher Scientific, Waltham, MA, USA, catalog No. 10270106). H358 (CRL-5807™) and H23 (CRL-5800™) cells were maintained in Roswell Park Memorial Institute 1640 Medium (RPMI 1640, GlutaMAX™ Supplement, Gibco by Thermofisher Scientific, Waltham, MA, USA, catalog No. 61870010) supplemented with 10% heat-inactivated low-endotoxin fetal bovine serum (FBS; Gibco by Thermofisher Scientific, Waltham, MA, USA, catalog No. 10270106). All cell media were ordinarily supplemented with antibiotics (100 U/mL penicillin and 100 ug/mL streptomycin; Gibco by Thermo Fisher Scientific, Waltham, MA, USA, catalog No. 15140122). Cells were maintained in a 37 °C and 5% CO2 air incubator and routinely screened for the absence of mycoplasma contamination.

### 2.2. Gene Silencing and Overexpression

Gene silencing was performed as explained in Fiorito et al. [6]. Briefly, *FLVCR1a* was specifically down-regulated with a shRNA (TRC Lentiviral pLKO.1 Human *FLVCR1* shRNA set RHS4533-NM_014053, clone TRCN0000059599; Thermo Fisher Scientific, Inc.) targeting the first exon of the human *FLVCR1* gene. For control cells, a pLKO.1 scramble shRNA was used. Gene overexpression was obtained as described in Fiorito et al. [6]. The *FLVCR1a* cDNA present in pCMV-SPORT6 vector (MGC human FLVCR1 sequence, clone 4417876, Horizon Discovery, Cambridge, UK, catalog No. MHS6278-202757940) and the Myc-tag sequence present in pcDNA3.1 [14]/myc-His B (Thermofisher Scientific, Waltham, MA, USA, catalog No. V85520) were cloned in frame in the pLVX-puro vector, a lentiviral expression vector with constitutively active human cytomegalovirus immediate early promoter and the puromycin resistance gene (Clontech Laboratories Inc. A Takara Bio Company, Mountain View, CA, USA, catalog number 632164). For control cells, a pLVX-puro empty vector (Clontech Laboratories Inc. A Takara Bio Company, Mountain View, CA, USA, catalog number 632164) was used.

Following lentiviral transduction, cells were maintained in selective medium containing 0.02 µg/mL puromycin.

### 2.3. RNA Extraction and Quantitative Real-Time-PCR Analysis

RNA extraction and quantitative real-time PCR analyses were performed as described previously [6]. Briefly, total RNA was extracted using Purelink micro to midi RNA extraction kit (Thermofisher Scientific, Waltham, MA, USA). Between 500 and 1000 ng of total RNA were transcribed into complementary DNA (cDNA) by High-Capacity cDNA Reverse Transcription Kit (Thermofisher Scientific, Waltham, MA, USA). Quantitative real-time PCR [15] was performed using the Universal Probe Library system (Roche, Basel, Switzerland). *ALAS1* primers (primer sequences: Left primer GAAATGAATGCCGTGAGGAA; Right primer CCTCCATCGGTTTTCACACT) and probe (probe #40; Roche, Basel, Switzerland, catalog No. 04687990001) were designed using the ProbeFinder software (www.roche-applied-science.com (accessed on 18 May 2018)). qRT-PCR were performed on QuantStudio 6 Flex Real-Time PCR System (Thermofisher Scientific, Waltham, MA, USA) and the analyses were done using QuantStudio 6 software. Transcript abundance, normalized to beta-actin (Thermofisher Scientific, Waltham, MA, USA, catalog No. 4310881E) mRNA expression, is expressed as a fold increase over a calibrator sample.

### 2.4. Western Blot

Western blot experiments were performed as described in [6]. Briefly, to assess FLVCR1a and ALAS1 expression, cells were lysed by rotation for 30 min at 4 °C in RIPA buffer (150 mM NaCl, 50 mM Tris-HCl pH 7.5, 1% Igepal NP-40, 0.5% Sodium deoxycholate, 0.1% SDS, 1 mM EDTA)_._ The buffer was freshly supplemented with 1 mM phosphatase inhibitor cocktail (Sigma Aldrich, St. Louis, MO, USA, catalog No. P0044), 1 mM PMSF (Sigma Aldrich, St. Louis, MO, USA, catalog No. 93482-50ML-F), and protease inhibitor cocktail (La Roche, Basel, CH, catalog No. 04693116001). The cell lysate was clarified by centrifugation for 10 min at 4 °C. Protein concentration in the supernatant was assessed by Bradford assay. For FLVCR1a detection, to remove protein glycosylation, 10 ug of protein extracts were incubated 10 min at 37 °C with 1 uL of PNGase-F from Elizabethkingia meningoseptica (Sigma Aldrich, St. Louis, MO, USA, catalog No. P-7367). Before loading on 4–15% mini-PROTEAN TGX precast gel (Bio-Rad, Hercules, CA, USA, catalog No. 4568084), samples were incubated 5 min at 37 °C (for FLVCR1a detection) or 5 min at 95 °C (for ALAS1 detection) in 4× laemmli buffer freshly supplemented with 8% 2-mercaptoethanol. The primary antibodies and dilutions are as follows: FLVCR1 (C-4) (Santa Cruz Biotechnology, Dallas, TX, USA, catalog No. sc-390100; 1:500), ALAS1 (Santa Cruz Biotechnology, Dallas, TX, USA, catalog No. sc-137093; 1:1000), beta-actin (Santa Cruz Biotechnology, Dallas, TX, USA, catalog No. sc-1616; 1:1000) and vinculin (home-made, 1:8000). The revelation was assessed using the ChemiDoc Imaging System (Bio-Rad, Hercules, CA, USA).

### 2.5. Cells Treatment with Drugs

5-Fluorouracil was purchased by Sigma Aldrich-Merck KGaA (Darmstadt, Germany catalogue No. F6627). Irinotecan and Oxaliplatin were purchased by Selleckchem (Selleck chemicals, Munich, Germany, catalogue No. S4908 and S1224, respectively). The three drugs were chosen as representative standard chemotherapeutics used in the therapy of colorectal cancer [16].

Stock solutions were prepared in dimethyl sulfoxide (DMSO) and then diluted to the desired concentrations in culture medium. DMSO final concentration was kept lower than 0.1% to avoid DMSO-induced toxicity.

Metformin (Sigma Aldrich by Merck KGaA, Darmstadt, Germany, catalogue No. PHR1084-500MG) was freshly prepared in culture medium. Drugs were administered 12 h after cells plating. 7 uM 5-FU, 50 nM SN38, 5 uM Oxaliplatin, 2.5 mM Metformin doses were chosen according to the literature data [14,17,18,19]. Cells were exposed to the drug the day after plating and maintained without medium refresh for 24, 48 or 72 h.

### 2.6. Evaluation of Cell Proliferation

H358 cells proliferation was assessed by manual count. Briefly, 300,000 cells/well were plated in 6-well plates. After 1 and 2 days of drug administration, cells were trypsinized and counted using a Neubauer improved cell counting chamber.

For the other cell lines, cell viability was measured at different time points as a readout of cell proliferation. To assess it, 10,000 SKCO1, 3000 PANC, 4000 HPAC, 5000 H23 and 5000 CACO2 cells/well were plated in their culture medium (or, for CACO2 cells, in culture medium but with only 10% FBS instead of 20% FBS) and analyzed with the Cell Titer-Fluor™ Cell Viability Assay (Promega, Madison, WI, USA, catalogue No. G6080) according to manufacture instructions.

### 2.7. Crystal Violet Staining

Experiments were performed as described in [6]. CACO2 cells were plated in 12-well plates (1500 cells/well) and stained after 1 and 2 days of drug administration.

### 2.8. Mitochondria Isolation and ETC Complexes Activity Measurment

According to Wibom et al. [20], cells were washed twice in ice-cold 0.1 M phosphate-buffered saline (PBS), then lysed in 0.5 mL buffer A (50 mmol/L Tris, 100 mmol/L KCl, 5 mmol/L MgCl_2_, 1.8 mmol/L ATP, 1 mmol/L EDTA, pH 7.2), supplemented with protease inhibitor cocktail III [100 mmol/L AEBSF, 80 mmol/L aprotinin, 5 mmol/L bestatin, 1.5 mmol/L E-64, 2 mmol/L leupeptin and 1 mmol/ L pepstatin (Merck KGaA, Darmstadt, DE)], 1 mmol/L phenylmethylsulfonyl fluoride (PMSF), 250 mmol/L NaF. Samples were clarified by centrifuging at 650× *g* for 3 min at 4 °C, and the supernatant was collected and centrifuged at 13,000× *g* for 5 min at 4 °C. The new supernatant was discarded, the pellet containing mitochondria was washed in 0.5 mL buffer A and re-suspended in 0.25 mL buffer B (250 mmol/L sucrose, 15 mmol/L K_2_HPO_4_, 2 mmol/L MgCl_2_, 0.5 mmol/L EDTA, 5% *w*/*v* bovine serum albumin). Afterwards, the activity of mitochondria respiration complexes was measured according to Wibom et al. [20].

### 2.9. Statistical Analyses

Statistical comparisons were conducted in Prism (GraphPad Software, Inc., La Jolla, CA, USA), using one-way or two-way ANOVA followed by Bonferroni correction for multiple groups comparison. An unpaired Student’s *t*-test was used when only two groups were compared. A *p*-value of less than 0.05 was regarded as significant.

## 3. Results

### 3.1. FLVCR1a Silencing Improves Metformin Effect on CRC Cell Lines

**Figure 1:**
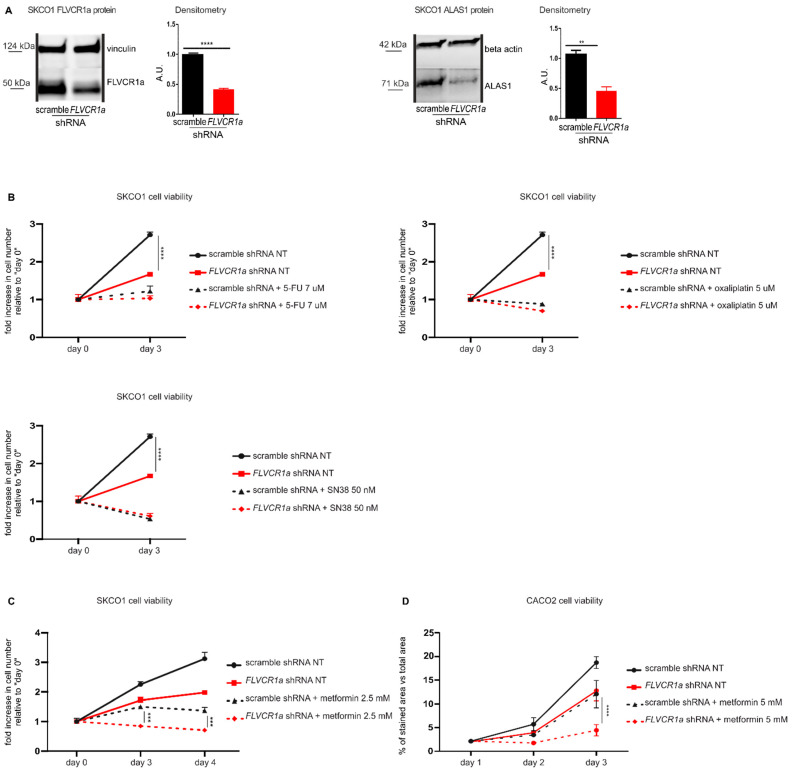
The down-modulation of the heme synthesis-export system, obtained by *FLVCR1a* silencing, sensitizes colorectal cancer cells to metformin. (**A**)Western blot analysis of FLVCR1a and ALAS1 expression in SKCO1 cells. Vinculin and beta-actin expression are shown as a loading control, respectively. Cells in which the expression of *FLVCR1a* was down-regulated using a specific shRNA are compared to cells expressing a scramble shRNA. A representative blot is shown. For statistical analyses, a Student *t*-test was used. Densitometry shows mean ± SEM, *n* = 3 (FLVCR1a blot) and 2 (ALAS1 blot) biological replicates, ** *p* < 0.01, **** *p* < 0.0001. (**B**,**C**) Cell viability of SKCO1 cells untreated (NT) or treated with 7 uM 5-FU, 5 uM Oxaliplatin and 50 nM SN38 (**B**) or with 2.5 mM metformin (**C**), measured after two days (5-FU, Oxaliplatin and SN38) or two and three days (metformin) of drug exposure without medium refresh. Cells in which *FLVCR1a* expression was down-modulated using a specific shRNA are compared to cells expressing a scramble shRNA. Cell viability is measured as a fold increase over an untreated calibrator sample and expressed relative to day 0. For statistical analyses a two-way analysis of variance was performed, followed by the Bonferroni correction for multiple groups comparisons. Values represent mean ± SEM, *n* = 4 biological replicates, *** *p* < 0.001, **** *p* < 0.0001. (**D**) Cell viability of CACO2 cells was assessed by crystal violet staining on the indicated days. Cells in which the expression of *FLVCR1a* was down-regulated using a specific shRNA are compared to cells expressing a scramble shRNA; 5 mM metformin administration occurred at day 1 of cell proliferation and medium was maintained without refresh till the end of the experiment. Staining quantification is reported in the graph as the percentage of crystal violet stained area *versus* image total area. For statistical analyses, a two-way analysis of variance was performed, followed by the Bonferroni correction for multiple groups comparisons. Values represent mean ± SEM, *n* = 3 biological replicates, **** = *p* < 0.0001.

To assess whether the inhibition of the heme synthesis-export system affects the sensitivity of tumor cells to anticancer therapies, we silenced *FLVCR1a* first in SKCO1 cells, a colorectal cancer cell line. The down-modulation of *FLVCR1a* in these cells resulted in reduced ALAS1 expression (Figure 1A and Figure S2A,B) confirming that the block of heme efflux inhibits heme synthesis. As we previously reported, the inhibition of the heme synthesis-export system in these cells, as in other cell lines, promotes oxidative metabolism and impairs cell proliferation both in vitro and in vivo [6].

We evaluated the response of *FLVCR1a*-silenced SKCO1 cells to 5-Fluorouracil (5-FU), Oxaliplatin, Irinotecan (SN38) and metformin. 5-FU, Oxaliplatin and Irinotecan are standard chemotherapeutics for the treatment of CRC [16], while metformin is an antidiabetic drug with anticancer properties. 5-FU, Oxaliplatin and SN38 treatments were not affected by *FLVCR1a* silencing (Figure 1B). Conversely, *FLVCR1a* down-modulation significantly improved the effect of metformin at 2.5 mM (Figure 1C), 5 and 10 mM (Supplementary Figure S1A,B) doses, as silenced cells showed significantly reduced cell viability as compared to treated control cells (Figure 1C).

In order to confirm the data obtained in SKCO1 cells in another colorectal cancer cell line, we analyzed *FLVCR1a*-silenced CACO2 cells. Similarly to SKCO1 cell line, silencing of *FLVCR1a* in CACO2 cells [6] (Supplementary Figures S1C and S2F) reduced cell proliferation (Figure 1D). Moreover, treatment with 5 mM metformin strongly reduced cell proliferation of *FLVCR1a*-silenced CACO2 cells as compared to treated controls (Figure 1D). Conversely, overexpression of *FLVCR1a* (Supplementary Figures S1D and S2G) notably increased cell proliferation compared to controls. In addition, although the effect was mild, we detected a statistically significant prevention of 5 mM metformin inhibitory effect on the proliferation of *FLVCR1a* overexpressing cells (Supplementary Figure S1E).

These data indicate that the inhibition of FLVCR1a-mediated heme efflux improves the effect of metformin on CRC cell lines, thus rendering this combined approach a potential valuable strategy to reduce tumor cell proliferation.

### 3.2. ALA Treatment Improves Metformin Effect on CRC Cell Lines

The results obtained support the combination of metformin with the targeting of the heme synthesis-export system to boost the anti-proliferative effect.

To phenocopy *FLVCR1a* down-modulation with a clinically applicable compound, SKCO1 cells were treated with ALA. ALA is a heme precursor which, bypassing ALAS1, stimulates heme biosynthesis and induces heme accumulation, leading to a feedback down-modulation of ALAS1 expression and activity. Therefore, ALA is expected to downmodulate the heme synthesis-export system, analogously to what observed with *FLVCR1a* silencing.

**Figure 2:**
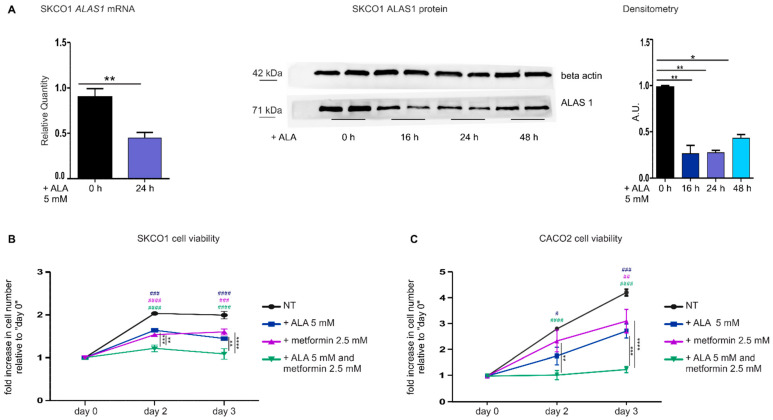
The down-modulation of the heme synthesis-export system, obtained by ALA treatment, sensitizes colorectal cancer cells to metformin. (**A**) qRT-PCR analysis (on the **left**) and Western blot analysis (on the **right**) of *ALAS1* expression in SKCO1 cells upon ALA administration. For qRT-PCR analysis, *ALAS1* expression was measured on SKCO1 cells untreated or treated with 5 mM ALA for 24 h. Transcript abundance is normalized on beta-actin mRNA expression and expressed as a fold increase over a calibrator sample. For statistical analyses, a Student-*t*-test was used. Data represent mean ± SEM, *n* = 5 biological replicates, ** *p* < 0.01. For western blot analysis, untreated cells are compared to cells treated with 5 mM ALA for 16, 24 and 48 h. Beta-actin expression is shown as a loading control. Band intensities were measured by densitometry and normalized to beta-actin expression. Densitometry data represent mean ± SEM, *n* = 2 biological replicates. For statistical analysis, a one-way ANOVA analysis of variance was performed, followed by the Bonferroni correction for multiple groups comparisons; * *p* < 0.05, ** *p* < 0.01. (**B**,**C**) Cell viability of SKCO1 (**B**) and CACO2 (**C**) cells measured at different time points as a readout of cell proliferation. Untreated (NT) cells are compared to cells treated with ALA, metformin or ALA + metformin. Drug administration occurred at day 1 of cell proliferation. Cell viability is measured as a fold increase over an untreated calibrator sample and expressed relative to day 0. Data represent mean ± SEM, *n* = 5 (SKCO1) or 4 (CACO2) biological replicates. For statistical analyses, a two-way analysis of variance was performed, followed by the Bonferroni correction for multiple groups comparisons; * symbols are used to indicate statistical significance when cells treated with ALA + metformin are compared to cells treated with metformin or ALA alone; # symbols are used to indicate statistical significance when treated cells are compared to untreated cells; # *p* < 0.05, ** or ## *p* < 0.01, *** or ### *p* < 0.001, **** or #### *p* < 0.0001.

Upon exposure to 5 mM ALA for 16, 24 and 48 h, *ALAS1* mRNA and protein levels strongly decreased in SKCO1 cells, confirming the inhibitory effect of accumulated heme on ALAS1 (Figure 2A and Figure S2C). Moreover, 5 mM ALA treatment alone strongly reduced SKCO1 cell proliferation (Figure 2B), similarly to what observed with *FLVCR1a* silencing. Finally, ALA treatment improved SKCO1 cells sensitivity to 2.5 mM metformin (Figure 2B).

Similar results were obtained analyzing ALA-treated CACO2 cells (Figure 2C).

We concluded that the inhibition of the heme synthesis-export system, either by blocking heme export or by reducing heme synthesis, increases the sensitivity of CRC cells to metformin.

### 3.3. The Down-Modulation of the Heme Synthesis-Export System Improves Metformin Effect on Different Kinds of Tumor Cell Lines

**Figure 3:**
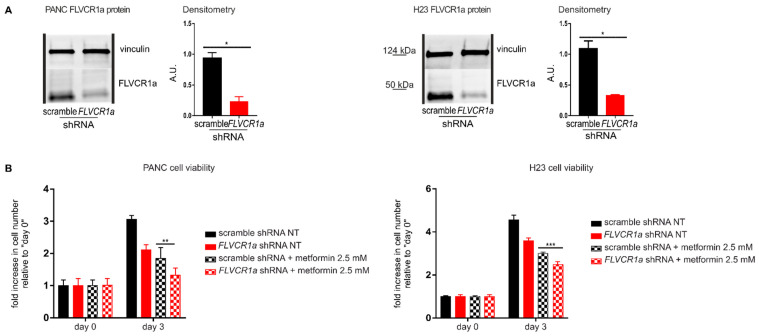

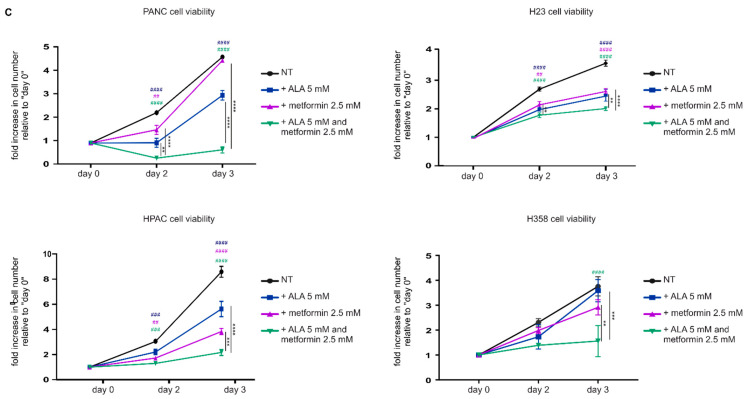
The down-modulation of the heme synthesis-export system sensitizes pancreatic and lung cancer cells to metformin. (**A**) Western blot analysis of FLVCR1a expression in PANC (on the **left**) and H23 (on the **right**) cells. Vinculin expression is used as a loading control. Cells in which the expression of *FLVCR1a* was down-regulated using a specific shRNA are compared to cells expressing a scrambled shRNA. A representative blot is shown. For statistical analyses a Student *t*-test was used. Densitometry shows mean ± SEM, *n* = 2 biological replicates, * *p* < 0.05. (**B**) Cell viability of PANC (on the **left**) and H23 (on the **right**) cells untreated (NT) or treated with 2.5 mM metformin, measured after two days of drug exposure without medium refresh. Cells in which *FLVCR1a* expression was down-modulated using a specific shRNA are compared to cells expressing a scramble shRNA. Cell viability is measured as a fold increase over an untreated calibrator sample and expressed relative to day 0. For statistical analyses, a two-way analysis of variance was performed, followed by the Bonferroni correction for multiple groups comparisons. Values represent mean ± SEM, *n* = 4 biological replicates, ** *p* < 0.01, *** *p* < 0.001. (**C**) Cell viability of PANC, HPAC H23 and H358 cells measured at different time points as a readout of cell proliferation. Untreated (NT) cells are compared to cells treated with ALA, metformin or ALA + metformin. Drug administration occurred at day 1 of cell proliferation. Cell viability of PANC, HPAC, and H23 was measured with Cell Titer-Fluor™ Cell Viability Assay as a fold increase over an untreated calibrator sample and expressed relative to day 0. Data represent mean ± SEM, *n* = 4 biological replicates. H358 cell proliferation was assessed with a manual count, data represent mean ± SEM, *n* = 2 biological replicates. For statistical analyses, a two-way analysis of variance was performed, followed by the Bonferroni correction for multiple groups comparisons; * symbols are used to indicate statistical significance when cells treated with ALA + metformin are compared to cells treated with metformin or ALA alone; # symbols are used to indicate statistical significance when treated cells are compared to untreated cells; ** or ## *p* < 0.01, *** or ### *p* < 0.001, **** or #### *p* < 0.0001.

Seminal works have highlighted the crucial role of heme metabolism in pancreatic cancer and lung cancer [21,22,23]. Moreover, metformin applicability to counteract pancreatic and lung cancer has already been proposed [24,25,26,27]. In order to verify whether the sensitizing effect of the inhibited heme synthesis-export system to metformin is restricted to CRC cells or can be extended to other kinds of tumor cells, we moved to the analyses of pancreatic cancer (PANC) and lung cancer (H23) cell lines. Upon *FLVCR1a* silencing (Figure 3A and Figure S2D,E), we detected decreased cell growth (Figure 3B). In addition, we observed increased sensitivity to metformin (Figure 3B), confirming the data obtained in CRC cell lines.

Then, we tested the alternative approach: the administration of ALA. ALA-treated pancreatic (PANC and HPAC) and lung (H23 and H358) cancer cells showed decreased cell proliferation and increased sensitivity to metformin (Figure 3C), again recapitulating the results achieved in CRC cells.

**Figure 4:**
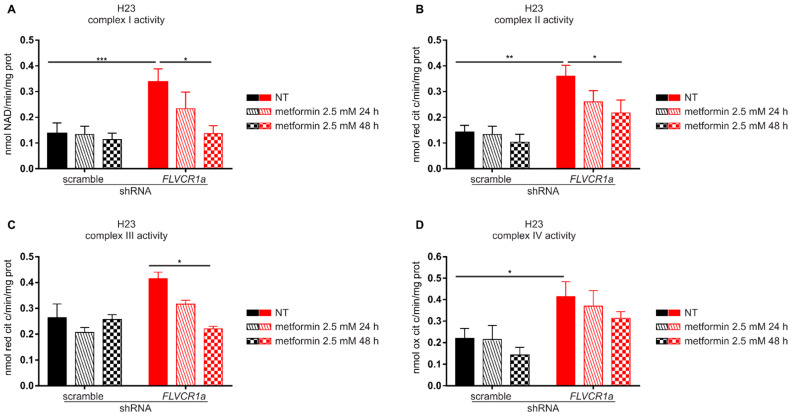
Metformin decreases ETC complexes activity in *FLVCR1a*-silenced cells. Activities of the mitochondrial electron transport chain complexes I–IV in H23 cells untreated (NT) and treated with 2.5 mM metformin for 24 or 48 h. *FLVCR1a*-silenced H23 cells are compared with cells expressing a scramble shRNA. Results were expressed as nmol of NAD+/min/mg of mitochondrial protein for complex I (**A**), nmol of reduced cytochrome c/min/mg of mitochondrial protein for complexes II–III (**B**,**C**), and nmol of oxidized cytochrome c/min/mg of mitochondrial protein for complex IV (**D**). Data represent means ± SEM, *n* = 3. For statistical analyses, a two-way analysis of variance was performed, followed by the Bonferroni correction for multiple groups comparisons * *p* < 0.05, ** *p* < 0.01, *** *p* < 0.001.

Metformin is known to affect several cellular processes and to exert its anti-proliferative effect through different mechanisms [28,29]. Among them, metformin is a recognized ETC-interfering agent [30]. The disruption of the heme synthesis-export axis is associated to increased ETC complexes activity in several cell lines [6]. Therefore, we postulated that the increased vulnerability to the anti-proliferative effects of metformin observed in *FLVCR1a*-silenced cells could be ascribed to the enhanced dependence of these cells to OXPHOS. To test this hypothesis, we analyzed the activity of ETC complexes in *FLVCR1a*-silenced H23 cells treated with the same dose of metformin that was used to inhibit cell growth. We detected increased ETC complexes activity in *FLVCR1a*-silenced cells relative to controls (Figure 4), in agreement with data we previously reported in other cell lines [6]; moreover, we observed that while metformin, at the dose used, did not significantly affect ETC complexes activities in control cells, it dramatically decreased them in *FLVCR1a*-silenced cells (Figure 4). These results suggest that, while the anti-proliferative action of metformin in *FLVCR1a* expressing cells relies, at the doses used, on ETC-independent effects, in *FLVCR1a*-silenced cells, it could be based, at least in part, on ETC inhibition.

Overall, the data confirm the requirement of a functional heme synthesis-export axis to sustain cell proliferation and provide compelling evidence that disruption of this system, by either RNA-interference technology or by a pharmacological approach, sensitizes tumor cells to metformin anti-proliferative effect, likely by enhancing cell vulnerability to metformin-mediated ETC inhibition. Finally, the results support the combination of ALA with metformin, as a promising clinically applicable approach to possibly counteract different kinds of tumors.

## 4. Discussion

Cancer is a leading cause of mortality worldwide. Many innovative therapeutic opportunities are available to target neoplasia; however, the onset of drugs resistance as well as the side effects related to some of these treatments, are still a main cause of poor patients’ survival. The oral antihyperglycemic drug metformin appears very promising for the treatment of different kinds of tumors [31]: several studies reported its efficacy in suppressing lung and pancreatic cancer progression [24,25,26,27], as well as colorectal aberrant crypt foci growth [32], and in ameliorating the survival of diabetic patients affected by different kinds of tumors, including lung cancer [33,34], pancreatic cancer [35,36,37,38] and CRC [35,39]. The efficacy of metformin is associated with safety [40]. Therefore, drug repurposing for metformin appears a concrete option.

The rewiring of heme metabolism has emerged as a crucial feature of tumor cells [2]. Particularly, heme synthesis and degradation represent metabolic dependencies in pancreatic/lung cancer [21] and hereditary leiomyomatosis and renal-cell cancer [8], respectively. Moreover, we recently reported that a functional relationship between heme synthesis and heme export sustains the reshaping of cellular energy metabolism necessary for CRC cells proliferation and survival [6]. Finally, several works reported changes in cancer cell response to antitumor treatment upon de-regulation of heme homeostasis [7,9,10,11,12,13].

Here, we show that metformin anti-proliferative effect on CRC cell lines, as well as on lung and pancreatic cancer cell lines, is improved by the inhibition of the heme synthesis-export system.

Although the precise mechanism responsible for the effects observed requires further investigation, some hypotheses can be formulated. Metformin exerts an anticancer activity through different mechanisms [28,29]. One of them relies on the blockage of the electron transport chain (ETC) complex I. The consequent drop of ATP levels activates the energy sensor protein AMPK, which directly inhibits mTOR, a complex involved in cell proliferation control [29]. In addition, metformin has also an ETC-independent action. Indeed, it has been reported to cause LKBI-mediated AMPK activation, leading to the inhibition of PI3K/AKT pathway and to the reduction of pro-tumorigenic de novo lipid biosynthesis [28,29]. Furthermore, metformin was described to induce Bcl2 family-cytochrome c mediated apoptosis and to target mitochondrial integrity by regulating calcium flux in cancer cells [28]. Both ETC-related and ETC-unrelated mechanisms result in decreased cell proliferation and angiogenesis, thus reducing the growth potential of cancer cells [28,29]. As stated above, we have recently showed that, in SKCO1 and CACO2 cells, as well as in other cancer cell lines, the inhibition of heme export results in a transient accumulation of heme that negatively regulates ALAS1-mediated heme synthesis. This is associated with enhanced TCA cycle flux and increased activity of ETC complexes [6]. Moreover, here we demonstrate that the same inhibition of heme export enhances cell responsiveness to metformin-mediated ETC suppression. Therefore, collectively these data suggest that the dependency on OXPHOS of tumor cells with a disrupted heme synthesis-export axis might be the cause of their increased vulnerability to metformin anti-proliferative effect, likely through its ETC-related mechanism of action.

These data agree with those reported by Lee et al. [7] showing that BACH1 degradation by hemin sensitizes triple negative breast cancer cells to metformin. Indeed, BACH1 decreases glucose utilization in the TCA cycle, and it negatively regulates transcription of ETC genes. Thus, it is possible that both mechanisms, heme-mediated BACH1 degradation and heme-mediated ALAS1 inhibition, contribute to enhance cell sensitivity to metformin.

Independently of the underlying mechanism, the data reported in the present work indicate that the inhibition of the heme synthesis-export system, by either RNA-interference technology or by a pharmacological approach, could represent a valuable strategy to sensitize tumor cells to metformin anti-proliferative effect.

In addition, another interesting aspect emerged from our findings: our data show that ALAS1 inhibition by ALA has a cytostatic effect on tumor cells, by inhibiting cell proliferation similarly to FLVCR1a-mediated heme export blockage. These data further strengthen the concept of a deep relationship between ALAS1 and FLVCR1a, demonstrating that the disruption of the ALAS1-FLVCR1a system, either by blocking heme synthesis or by interfering with heme export, raises similar effects on cell growth. The identification of two alternative methods to efficiently disrupt the heme synthesis-export system is extremely relevant for possible future therapeutic application of these discoveries in different kinds of tumors. In addition, our results unveil for the first time a cytostatic effect of ALA per se, as in previous works this drug was always used as a photosensitizing agent in photodynamic therapy or a sensitizer for other drugs. We do not know whether this effect is restricted to tumor cells with a specific profile of heme metabolism, and further experiments are required to test its efficacy in vivo; however, the discovery is promising and puts forth the basis for future studies aimed at understanding the potential use of ALA as an antitumor agent per se.

Finally, our experiments showed that cells treated with ALA are more sensitive to metformin. This could be explained by the expected increased OXPHOS resulting from the block of the heme synthesis-export system induced by ALA treatment. In addition to this hypothesis, considering the cytostatic effect raised by ALA per se, we cannot exclude the possibility that decreased cell proliferation upon metformin-ALA combined treatment is due to metformin-mediated sensitization to ALA, rather than the opposite. Further experiments are required to better elucidate the mechanism responsible for the synergistic effects of the two drugs.

## 5. Conclusions

In conclusion, our work indicates that interfering with the ALAS1-FLVCR1a axis represents a valuable strategy to modulate tumor cell metabolism and improve sensitivity to specific drugs. These discoveries could potentially contribute to identify new future therapeutic strategies against cancer.

## 6. Patents

The University of Turin filed a patent application (No. 102021000015368_11th June 2021), whose inventors are A.L.A., F.B., S.P., D.C., V.F. and E.T., with data included in this manuscript.

## Data Availability

The data presented in this study are available on request from the corresponding author.

## Supplementary Materials

The following supporting information can be downloaded at: https://www.mdpi.com/article/10.3390/cancers14051230/s1, Figure S1. The modulation of the heme synthesis-export system, obtained by *FLVCR1a* silencing or overexpression alters colorectal cancer cells response to metformin. (A,B) Cell viability of SKCO1 cells untreated (NT) or treated with 5 mM (A) or 10 mM metformin (B), measured after two and three days of drug exposure without medium refresh. Cells in which *FLVCR1a* expression was down-modulated using a specific shRNA are compared to cells expressing a scramble shRNA. Cell viability is measured as a fold increase over an untreated calibrator sample and expressed relative to day 0. For statistical analyses a two-way analysis of variance was performed, followed by the Bonferroni correction for multiple groups comparisons. Values represent mean ± SEM, *n* = 4 biological replicates, * *p* < 0.05, *** *p* < 0.001, **** *p* < 0.0001. (C,D) Western blot analysis of FLVCR1a expression in CACO2 cells. Vinculin is shown as a loading control. Cells in which the expression of *FLVCR1a* was down-regulated using a specific shRNA are compared to cells expressing a scramble shRNA (C), while cells in which the expression of *FLVCR1a* was up-regulated using a specific lentiviral vector are compared to cells stably transduced with an empty vector (D). A representative blot is shown. For statistical analyses a Student-t test was used. Densitometry shows mean ± SEM, *n* = 2 biological replicates, * *p* < 0.05, ** *p* < 0.01. (E) Cell viability of CACO2 cells untreated (NT) or treated with 5 mM metformin, measured after three days of drug exposure without medium refresh. Cells in which *FLVCR1a* was overexpressed using a specific lentiviral vector are compared to cells stably transduced with an empty vector. Cell viability is measured as a fold increase over an untreated calibrator sample and expressed relative to day 0. For statistical analyses a two-way analysis of variance was performed, followed by the Bonferroni correction for multiple groups comparisons. Values represent mean ± SEM, *n* = 4 biological replicates, * *p* < 0.05, **** *p* < 0.0001. Figure S2 Western blot analysis of FLVCR1a and ALAS1 in SKCO1, PANC, H23 and CACO2 cell lines. (A,D,E,F,G) Western blot analysis of FLVCR1a expression in SKCO1 (A), PANC (D), H23 (E) and CACO2 (F,G) cells. Vinculin is shown as a loading control. Cells in which the expression of *FLVCR1a* was down-regulated using a specific shRNA are compared to cells expressing a scramble shRNA (A,D,E,F), while cells in which the expression of *FLVCR1a* was up-regulated using a specific lentiviral vector are compared to cells stably transduced with an empty vector (G). A representative blot is shown. On the right, band intensity of both FLVCR1a and Vinculin are reported, together with ratio and calibration values used in the densitometry analysis. (B) Western blot analysis of ALAS1 expression in SKCO1 cells. Beta actin is shown as a loading control. Cells in which the expression of *FLVCR1a* was down-regulated using a specific shRNA are compared to cells expressing a scramble shRNA. A representative blot is shown. On the right, band intensity of both ALAS1 and beta actin are reported, together with ratio and calibration values used in the densitometry analysis. (C) Western blot analysis of ALAS1 expression in SKCO1 cells. Beta actin is shown as a loading control. Untreated wild-type cells are compared to cells treated with 5 mM ALA for 16, 24 and 48 hours. On the right, band intensity of both ALAS1 and beta actin are reported, together with ratio and calibration values used in the densitometry analysis.
