# Supplementary material for: Inhibition of Heme Export and/or Heme Synthesis Potentiates Metformin Anti-Proliferative Effect on Cancer Cell Lines
Source: Cancers (Basel). 2022 Feb 27;14(5):1230. doi: 10.3390/cancers14051230 (PMC8908972; doi:10.3390/cancers14051230)
Supplement: Supplementary file 1 [file cancers-14-01230-s001.zip › cancers-1515920-supplementary.pdf]

Supplementary Materials

FIGURE S1

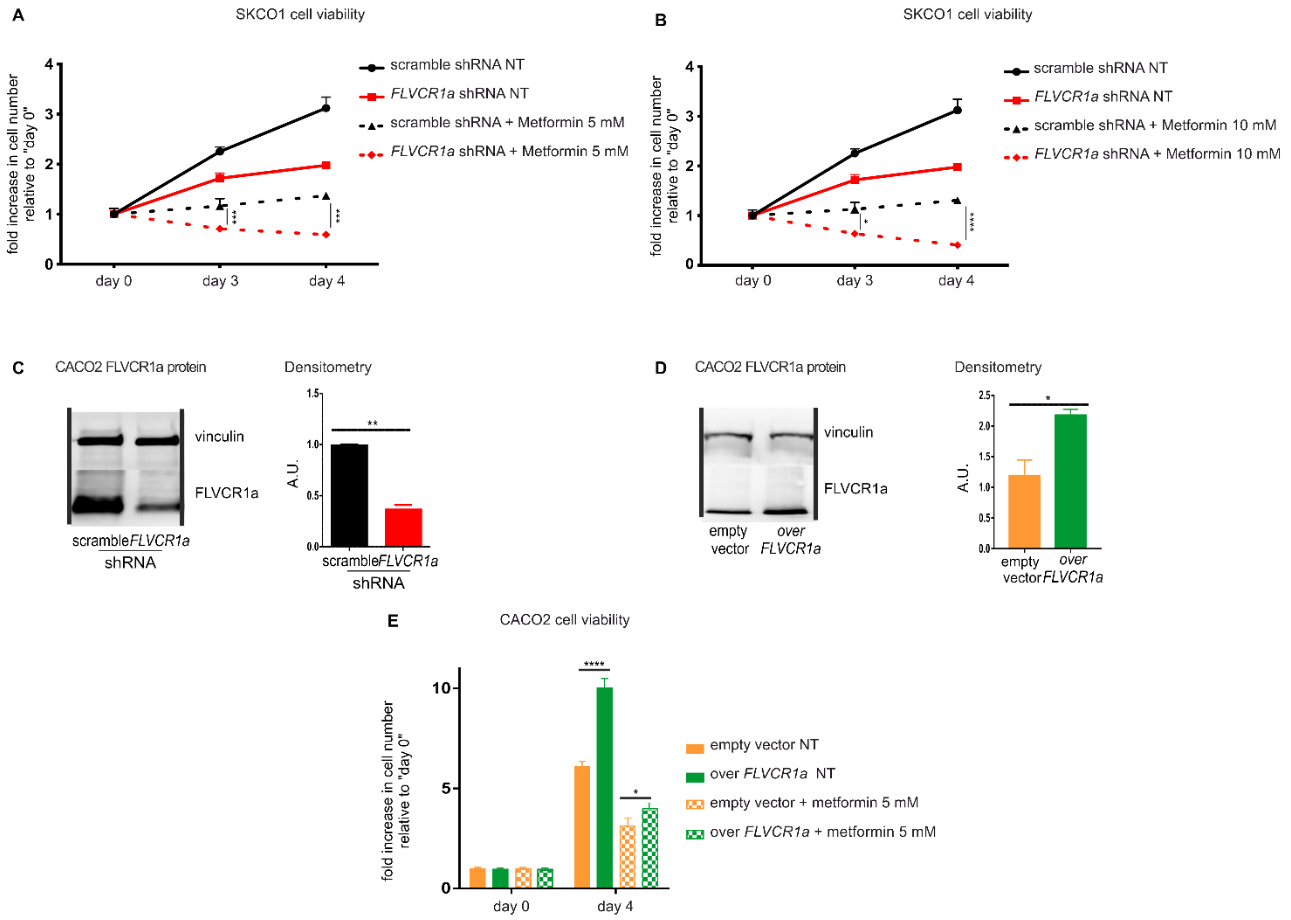

Figure S1. The modulation of the heme synthesis-export system, obtained by *FLVCR1a* silencing or overexpression alters colorectal cancer cells response to metformin. (A, B) Cell viability of SKCO1 cells untreated (NT) or treated with 5 mM (A) or 10 mM metformin (B), measured after two and three days of drug exposure without medium refresh. Cells in which *FLVCR1a* expression was down-modulated using a specific shRNA are compared to cells expressing a scramble shRNA. Cell viability is measured as a fold increase over an untreated calibrator sample and expressed relative to day 0. For statistical analyses a two-way analysis of variance was performed, followed by the Bonferroni correction for multiple groups comparisons. Values represent mean ± SEM, n=4 biological replicates, *=P<0.05, ***=P<0.001, ****=P<0.0001. (C, D) Western blot analysis of FLVCR1a expression in CACO2 cells. Vinculin is shown as a loading control. Cells in which the expression of *FLVCR1a* was down-regulated using a specific shRNA are compared to cells expressing a scramble shRNA (C), while cells in which the expression of *FLVCR1a* was up-regulated using a specific lentiviral vector are compared to cells stably transduced with an empty vector (D). A representative blot is shown. For statistical analyses a Student-t test was used. Densitometry shows mean ± SEM, n=2 biological replicates, *=P<0.05, **=P<0.01. (E) Cell viability of CACO2 cells untreated (NT) or treated with 5 mM metformin, measured after three days of drug exposure without medium refresh. Cells in which *FLVCR1a* was overexpressed using a specific lentiviral vector are compared to cells stably transduced with an empty vector. Cell viability is measured as a fold increase over an untreated calibrator sample and expressed relative to day 0. For statistical analyses a two-way analysis of variance was performed, followed by the Bonferroni correction for multiple groups comparisons. Values represent mean ± SEM, n=4 biological replicates, *=P<0.05, ****=P<0.0001.

FIGURE S2

A SKCO1 FLVCR1a protein

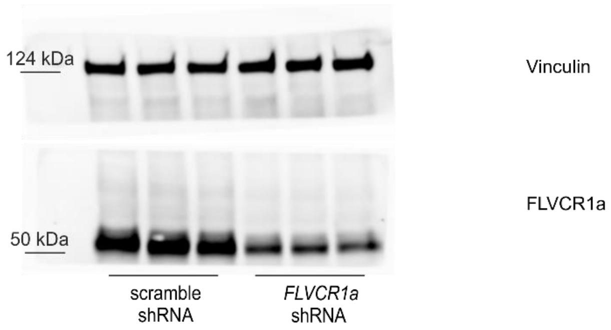

sample	band intensity FLVCR1a	band intensity Vinculin	ratio	calibration
scramble shRNA 1	131916739	40566754	3,25	1,00
scramble shRNA 2	131219323	38909838	3,37	1,04
scramble shRNA 3	112140888	36633320	3,06	0,94
FLVCR1a shRNA 1	63326830	44298708	1,43	0,44
FLVCR1a shRNA 2	60254831	43786187	1,38	0,42
FLVCR1a shRNA 3	61207944	49192311	1,24	0,38

B SKCO1 ALAS1 protein

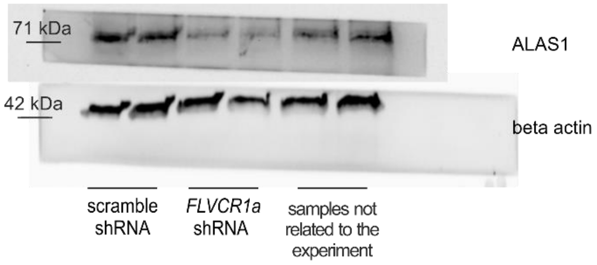

sample	band intensity ALAS1	band intensity beta actin	ratio	calibration
scramble shRNA 1	28539771	44380770	0,64	1,00
scramble shRNA 2	25997749	55227017	0,47	0,73
FLVCR1a shRNA 1	10729918	50334930	0,21	0,33
FLVCR1a shRNA 2	9420638	31912968	0,30	0,46

C SKCO1 ALAS1 protein

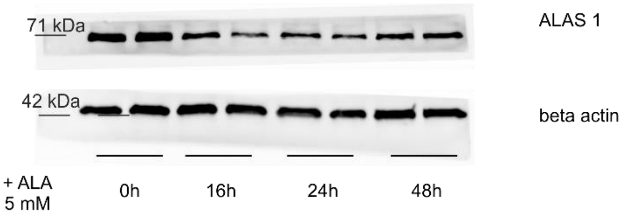

sample	band intensity Alas1	band intensity beta actin	ratio	calibration
SKCO1 + ALA 5mM 0h 1	24346210	33562308	0,73	1,00
SKCO1 + ALA 5mM 0h 2	28289830	40802161	0,69	0,96
SKCO1 + ALA 5 mM 16h 1	11022062	42807215	0,26	0,35
SKCO1 + ALA 5 mM 16h 2	4610164	36101678	0,13	0,18
SKCO1 + ALA 5mM 24h 1	7412778	34001760	0,22	0,30
SKCO1 + ALA 5mM 24h 2	4761131	26803283	0,18	0,24
SKCO1 + ALA 5mM 48h 1	11056390	39285757	0,28	0,39
SKCO1 + ALA 5mM 48h 2	12852016	37527343	0,34	0,47

D PANC FLVCR1a protein

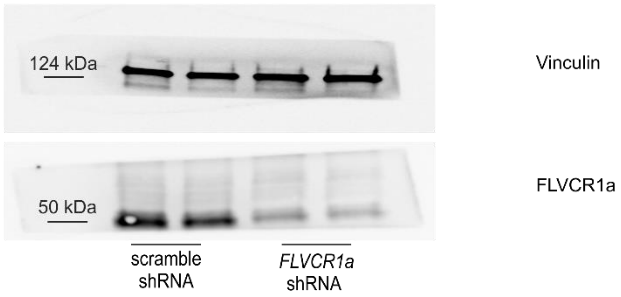

sample	band intensity FLVCR1a	band intensity vinculin	ratio	calibration
scramble shRNA 1	29668511	16126385	1,84	1,00
scramble shRNA 2	25134009	15688356	1,60	0,87
FLVCR1a shRNA 1	10826439	20588744	0,53	0,29
FLVCR1a shRNA 2	6362330	20239535	0,31	0,17

E H23 FLVCR1a protein

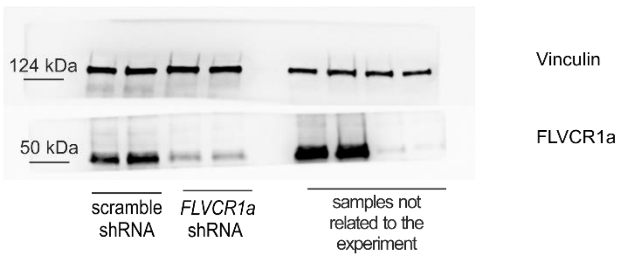

sample	band intensity FLVCR1a	band intensity vinculin	ratio	calibration
scramble shRNA 1	19848139	14571592	1,36	1,00
scramble shRNA 2	26723413	16608242	1,61	1,18
FLVCR1a shRNA 1	7582882	16408858	0,46	0,34
FLVCR1a shRNA 2	6729606	15583735	0,43	0,32

F CACO2 FLVCR1a protein

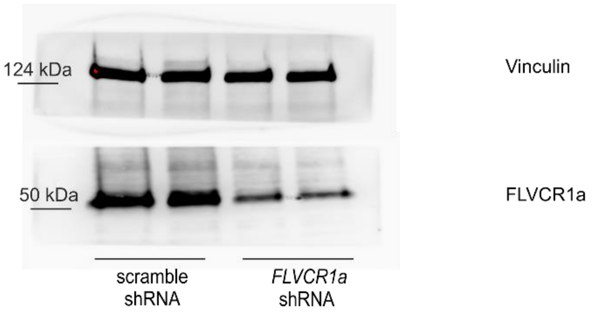

sample	band intensity FLVCR1a	band intensity vinculin	ratio	calibration
scramble shRNA 1	115411160	49981819	2,31	1,00
scramble shRNA 2	104404191	46261022	2,26	0,98
FLVCR1a shRNA 1	36827212	38831982	0,95	0,41
FLVCR1a shRNA 2	28582600	39843722	0,72	0,31

G CACO2 FLVCR1a protein

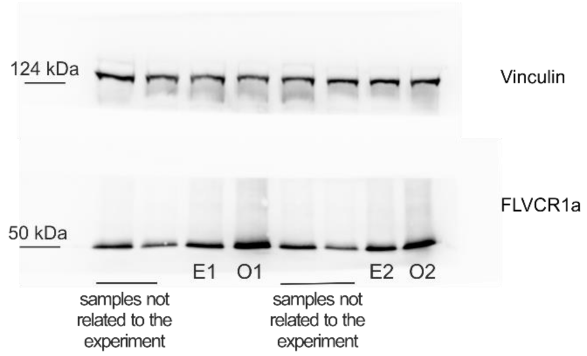

sample	band intensity FLVCR1a	band intensity vinculin	ratio	calibration
empty vector 1	64405278	16165378	3,98	1,00
empty vector 2	91353264	16722737	5,46	1,37
overFLVCR1a 1	140677787	15721565	8,95	2,25
overFLVCR1a 2	130511688	15573632	8,38	2,10

Legend
E= empty vector
O= over*FLVCR1a* vector

Figure S2 Western blot analysis of FLVCR1a and ALAS1 in SKCO1, PANC, H23 and CACO2 cell lines.

(A,D,E,F,G)Western blot analysis of FLVCR1a expression in SKCO1 (A), PANC (D), H23 (E) and CACO2 (F,G) cells. Vinculin is shown as a loading control. Cells in which the expression of *FLVCR1a* was down-regulated using a specific shRNA are compared to cells expressing a scramble shRNA (A,D,E,F), while cells in which the expression of *FLVCR1a* was up-regulated using a specific lentiviral vector are compared to cells stably transduced with an empty vector (G). A representative blot is shown. On the right, band intensity of both FLVCR1a and Vinculin are reported, together with ratio and calibration values used in the densitometry analysis. (B) Western blot analysis of ALAS1 expression in SKCO1 cells. Beta actin is shown as a loading control. Cells in which the expression of *FLVCR1a* was down-regulated using a specific shRNA are compared to cells expressing a scramble shRNA. A representative blot is shown. On the right, band intensity of both ALAS1 and beta actin are reported, together with ratio and calibration values used in the densitometry analysis. (C) Western blot analysis of ALAS1 expression in SKCO1 cells. Beta actin is shown as a loading control. Untreated wild-type cells are compared to cells treated with 5 mM ALA for 16, 24 and 48 hours. On the right, band intensity of both ALAS1 and beta actin are reported, together with ratio and calibration values used in the densitometry analysis.
